# A Review on the Detection of Plant Disease Using Machine Learning and Deep Learning Approaches

**DOI:** 10.3390/jimaging11100326

**Published:** 2025-09-23

**Authors:** Thandiwe Nyawose, Rito Clifford Maswanganyi, Philani Khumalo

**Affiliations:** 1 Department of Electronic and Computer Engineering, Durban University of Technology, Durban 4001, South Africa; 21711287@dut4life.ac.za; 2 Steve Biko Campus, Durban University of Technology, Durban 4001, South Africa; philanik1@dut.ac.za

**Keywords:** plant disease detection, image classification, machine learning, deep learning, convolutional neural networks, precision agriculture, image segmentation, real-time detection, YOLO, vision transformers, preprocessing techniques

## Abstract

The early and accurate detection of plant diseases is essential for ensuring food security, enhancing crop yields, and facilitating precision agriculture. Manual methods are labour-intensive and prone to error, especially under varying environmental conditions. Artificial intelligence (AI), particularly machine learning (ML) and deep learning (DL), has advanced automated disease identification through image classification. However, challenges persist, including limited generalisability, small and imbalanced datasets, and poor real-world performance. Unlike previous reviews, this paper critically evaluates model performance in both lab and real-time field conditions, emphasising robustness, generalisation, and suitability for edge deployment. It introduces recent architectures such as GreenViT, hybrid ViT–CNN models, and YOLO-based single- and two-stage detectors, comparing their accuracy, inference speed, and hardware efficiency. The review discusses multimodal and self-supervised learning techniques to enhance detection in complex environments, highlighting key limitations, including reliance on handcrafted features, overfitting, and sensitivity to environmental noise. Strengths and weaknesses of models across diverse datasets are analysed with a focus on real-time agricultural applicability. The paper concludes by identifying research gaps and outlining future directions, including the development of lightweight architectures, integration with Deep Convolutional Generative Adversarial Networks (DCGANs), and improved dataset diversity for real-world deployment in precision agriculture.

## 1. Introduction

Early disease detection promotes agricultural productivity as disease management strategies can be applied earlier. Farmers are less likely to apply pesticides indiscriminately at late growth stages, which allows the better control of crop production and increases economic output. According to a study focusing on the global burden of pathogens and pests on food security, 30% or more of current crop losses are attributed to undetected or untreated plant diseases [1]. Early detection assists in minimising crop losses, lowering pesticide expenses, as treatment is normally cheaper at early crop growth stages compared to late growth stages [2]. Farmers manually inspect the leaves for disease detection as this plant tissue displays signs of infection by either fungi, bacteria or viruses. Roots, stems, seeds, and fruits can also be inspected manually, as some diseases are easier to detect using these plant tissues [2].

Manual disease detection in plants is prone to human error due to the complexity and varying nature of plant leaves when they are infected [3]. Various diseases may occur at different growth stages or environmental conditions and exhibit similar symptoms, where even experienced farmers fail to identify the correct plant disease, thus leading to the incorrect administration of treatment to the plant [4]. These conventional detection methods focus on the colour, size and shape of the plant’s leaves, which is time-consuming as detection may require close inspection from the agricultural practitioner.

The introduction of Artificial Intelligence (AI) to plant disease detection enables faster disease detection through computer vision. Machine learning and deep learning reduced costs in terms of the number of experienced professionals required in crop fields for disease monitoring and detection. With the improvement of plant image datasets, precision agriculture improves as algorithms have more images to learn different patterns, thereby producing more accurate detection results and enhancing early disease detection and disease management.

The introduction of an automated disease detection system was to offer a solution for agricultural practitioners, such as farmers, agronomists and plant pathologists, to ensure accurate disease detection through plant leaves in a shorter detection period [3]. Automated detection of timely interventions allows farmers to implement disease treatment earlier or isolate the infected plant before the disease spreads to other plants, thus decreasing the intensity of infections and improving food security and sustainable farming practices [5].

The primary aim of this review paper is to investigate and critically evaluate the various approaches including models employed in the detection of plant diseases utilising machine learning (ML), and deep learning (DL) techniques.

This review aims to achieve the following objectives:

- To explore key topics in the use of ML and DL in plant disease detection, including various image preprocessing, segmentation techniques, feature extraction methods, and classification algorithms.
- Compare standard ML models (e.g., SVM, kNN, decision tree, Random Forest) with DL models (e.g., CNNs, AlexNet, VGGNet, ResNet, YOLO, ViTs, SSL and multimodal learning), detailing their performance across different crops and datasets, to conclude the suitability of models for various image datasets and natural environments.
- The paper also addresses the incorporation of hybrid models, the utilisation of specialised CNN architectures for specific crops, and the significance of model optimisation techniques such as transfer learning and ensemble learning.

The analysis of the findings will offer insight into future projects that aim to provide updated solutions for sustainable agricultural practices.

Key Contributions

This review presents a comprehensive analysis of CV-based ML and DL techniques for plant disease detection, emphasising real-time applications in uncontrolled agricultural environments. It highlights the key challenges of these techniques, compares classical and advanced models, and identifies future research directions for developing robust, field-ready systems.

- Discuss the limitations of traditional ML approaches for plant disease detection.
- Compares classical machine learning and deep learning models for plant disease detection.
- Reviews existing public datasets and their suitability for model training.
- Review models that address disease detection under the complex conditions of combined overlapping occlusion.
- Highlights limitations and suggests directions for robust, field-ready systems.
- Discuss the research gaps and provide insight for future studies.

Unlike prior reviews that focus solely on model performance under controlled conditions, this review uniquely emphasises how deep learning models, including ViTs and hybrid models, are deployed in controlled and natural agricultural environments. Highlighting each model’s suitability for robustness, generalizability and adaptability in varying environments. It critically evaluates how the model’s architecture affects its inference speed, hardware feasibility, and adaptability to class imbalance, and how it makes it a practical resource for researchers developing mobile or edge-based plant disease detection tools.

This paper is structured as follows: Section 2 discusses critical image preprocessing and segmentation techniques, as well as the biological and environmental elements that contribute to plant disease development. Section 3 introduces classical machine learning (ML) methods, evaluates their efficacy across various studies and discusses why some models perform better in controlled environments whilst others have low performance when trained on the same dataset. Section 4 describes the existing limitations of DL approaches in detail, focusing on traditional CNNs. Section 5 focuses on ViTs, SSL and multimodal learning. Lastly, Section 6 explores research gaps and applicable solutions to improve real-time plant disease detection.

### 1.1. Responsible Factors for Plant Diseases and Review Taxonomy

Plants whose physiological processes can be altered by environmental factors are receptive to diseases. Plant diseases are categorised into infectious diseases, which can be transmitted to healthy plants through the air or physical contact, and non-infectious diseases, where a single host is infected and the disease is not spread to other plants. Unfavourable growth conditions cause most non-infectious diseases, while infectious diseases are caused by biotic agents such as viruses, bacteria and fungi [6].

Table 1 displays different types of pathogens that affect plants with their respective symptoms. These pathogens are transmitted through mediums such as farm tools, wind and insects such as bees to other plants [6]. Rapid changes in climate change, environmental stressors and evolving farming practices also affect crop yield and the spread of disease [7]. Plants become easily susceptible to disease outbreaks, leading to crop losses and reduced agricultural quality in the surviving products.

**Figure 1:**
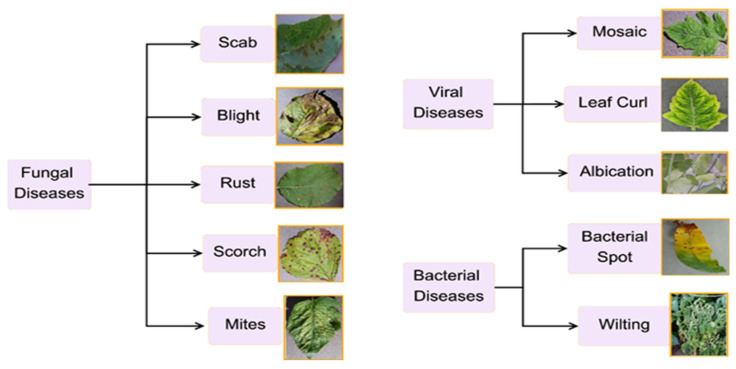
Pathogens and the infections they cause in various plants [8].

Figure 1 illustrates some of the infections that result from these pathogens, as listed in Table 1.

This review follows a narrative review approach aimed at synthesising current trends, limitations, and advancements in machine learning and deep learning methods for plant disease detection. To obtain the relevant papers for this study, a set of eligibility parameters was defined, and papers published on platforms such as Google Scholar, Elsevier, IEEE Xplore, ScienceDirect, and other scholarly databases up to 2025 were utilised. Commonly used search phrases, such as “plant disease detection”, “plant disease using deep learning”, “plant disease detection using machine learning”, and “deep learning” or “machine learning”, were employed.

### 1.2. Performance Evaluation

Model performances are commonly assessed using precision, F1 scores, recall, and accuracy rate. These metrics were computed by concatenating predictions across outer cross-validation folds and comparing them to the true labels. While accuracy in Equation (1) is an intuitive measure of model performance, as it illustrates the correctly classified samples to the total number of samples in the dataset, it becomes less reliable in the presence of class imbalance [8]. Therefore, most researchers also compute recall, as displayed in Equation (4), which is defined as the mean of the individual class accuracies. This is crucial in assessing the number of infected plants that were classified as healthy plants, allowing for early model optimisation to avoid infections from spreading.

(1) $$Accuracy=\frac{TP+TN}{FP+FN+TP+TN}$$

- TP—true positives, for correctly predicted positives.
- TN—true negatives, for correctly predicted negatives.
- FP—false positives, for incorrectly predicted positives.
- FN—false negatives, for incorrectly predicted negatives.

The model precision, as shown in Equation (2), captures the proportion of correctly predicted cases (true positives) among all predictions made (true positives + false positives). The F1 score in Equation (3), which represents the harmonic mean of precision and recall, was prioritised for model evaluation [8]. This metric is particularly appropriate in the presence of class imbalance and when it is crucial to optimise for both false positives and false negatives.

(2) $$Precision=\frac{TP}{FP+TP}$$

By balancing these two error types, the F1 score helps prevent the model from favouring one class over the other. In the scenario of plant disease detection, false negatives are arguably more critical than false positives, as they represent missed opportunities for treatment. However, false positives also warrant consideration due to time and resource constraints.

(3) $$F1\;Score=2\times \frac{Precision+Recall}{Precision\times Recall}$$

(4) $$Recall=\frac{TP}{TP+FN}$$

Therefore, while researchers primarily assess models based on the F1 score, they also report precision and recall to provide a comprehensive view of model behaviour, along with balanced accuracy as a secondary metric [8].

## 2. A Review on Plant Disease Image Preprocessing and Segmentation Techniques

**Figure 2:**
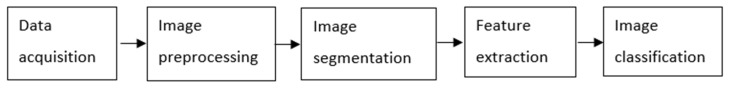
Conventional image processing pipeline for plant disease detection, including preprocessing, segmentation, feature extraction, and classification stages.

Extensive research and development efforts have been undertaken in recent years on plant disease detection through image preprocessing to improve the image data for training and testing while ensuring that no additional feature artefacts are added. These techniques ensure that there is sufficient brightness and removal of background artefacts in the image for effective detection. Figure 2 illustrates the conventional image processing technology stages used to identify plant diseases. This sequence forms the basis for image detection and has been adopted by various researchers for algorithm models.

In the early 2010s, one researcher used a camera to acquire images, which were then pre-processed and segmented before being subjected to the Gabor Filter for feature extraction. The Gabor filter analyses neighbouring pixels to detect texture changes in the leaf and the background, which also allows for edge detection [9]. Its accuracy rate improves when preprocessing steps have been applied, making the model efficient. Other basic preprocessing techniques include image resizing, data normalisation, colour space transformation (e.g., RGB to HSV), histogram equalisation, and noise reduction through Gaussian or median filtering [10].

Contrast Enhancement techniques were used to enhance the image quality by redistributing the pixel intensity using the transfer functions [11,12].

**Figure 3:**
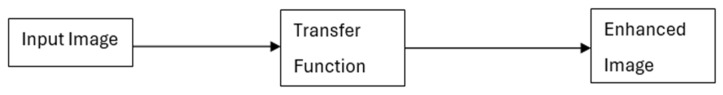
The flow diagram of how a transfer function is used to enhance an image.

Figure 3 illustrates how the transfer function is used to enhance the features of an image during preprocessing. The transfer function maps the original image pixel values to new values determined from the Cumulative Distribution Function (CDF) of the original image. The transfer function flattens the image histogram, resulting in a relatively uniform image with a clear distinction between varying features [13]. In plant disease detection, it makes it easier to differentiate the infected from the healthy portions in a leaf due to the uniform distribution of intensities. In a study of underwater image enhancements, a simplified homomorphic filter (HF) transfer function in Equation (5) was applied to suppress noise and enhance image contrast at specific frequency points [14].

(5) $$H\left(u,v\right)=\left(r_{H}-r_{L}\right)\left[1-e^{-c\left(\frac{D^{2}\left(u,v\right)}{D_{0}^{2}}\right)}\right]+r_{L}$$

where $r_{H}$ denotes the high frequency gain which controls how enhanced the fine textures and edges are, $r_{L}$ denotes the low frequency, which controls how much of the image illumination is kept, and Do denotes the cutoff frequency, which will determine where the filter will transition from low to high gain. C constant controls the sharpness or smoothness of the filter’s transition. Equation (1) displays various parameters that require extensive experiments for accurate use in a model. This creates a drawback as images in a dataset may require varying parameter values for accurate enhancement, which might be time-consuming for large datasets, or the image might be degraded and subjected to the removal of useful image information when the parameters are not well-tuned.

To further refine the image quality and address intensity imbalances, intensity normalisation is applied to scale the pixel values to a standard range of 0–255, where 0 is for black and 255 is for pure white. All the pixels in between this range are shades of grey. By normalising pixel intensities, the features affected by varying lightning and contrast levels are standardised. [13]. This enhances the robustness of the deep learning models where features such as leaf texture, lesion shape and colour variation are key indicators of infected and healthy plants.

**Figure 4:**
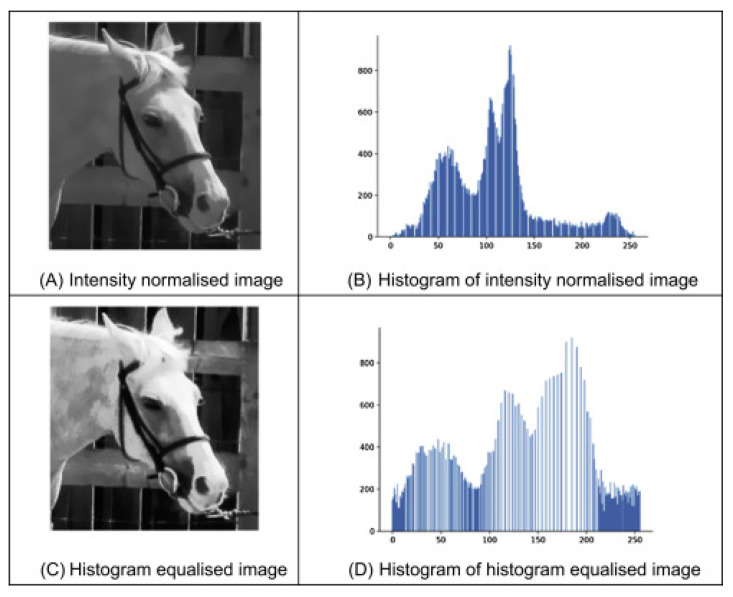
Histogram equalisation and image intensity normalisation in an image [13].

Figure 4 demonstrates the difference between an intensity-normalised image and a histogram equalised image, along with their corresponding histograms. Figure 4A shows the intensity-normalised image, which retains the overall structure while aligning the pixel values to a fixed scale [13]. Figure 4B presents the histogram of this image, where the distribution is still uneven, reflecting the original intensity variation. Figure 4C displays the histogram-equalised image, where the visual contrast has been significantly enhanced through histogram flattening. Figure 4D shows the corresponding histogram revealing an evenly distributed intensity across the pixel range [13]. This visual evidence highlights how histogram equalisation, through the transfer function, amplifies contrast and detail, making critical features in an image more discernible an essential step for precision in object classification tasks using machine vision techniques.

Further evidence of effective preprocessing comes from a study by researchers in [15] where basic image resizing and Gaussian filtering for noise reduction are applied to RGB images. The filtered images are converted to the HSV colour space to improve the separation of intensity and colour information. K-means clustering was used for segmentation, followed by a comparative analysis of classifiers. Among the evaluated models, CNN achieved superior performance with a classification accuracy of 98%, significantly outperforming traditional methods such as Logistic Regression (66.4%), KNN (54.5%), and SVM (53.4%) [15]. This underscores the effectiveness of traditional image preprocessing techniques when integrated with deep learning in capturing complex disease patterns. The adoption of CNN techniques also introduces the detection of more diseases in plants and mobile platforms deployments due to their lightweight architecture. However, this also introduces drawbacks; the high accuracy of the model is dependent on proper hyperparameter tuning, which requires experimentation and domain expertise.

**Figure 5:**
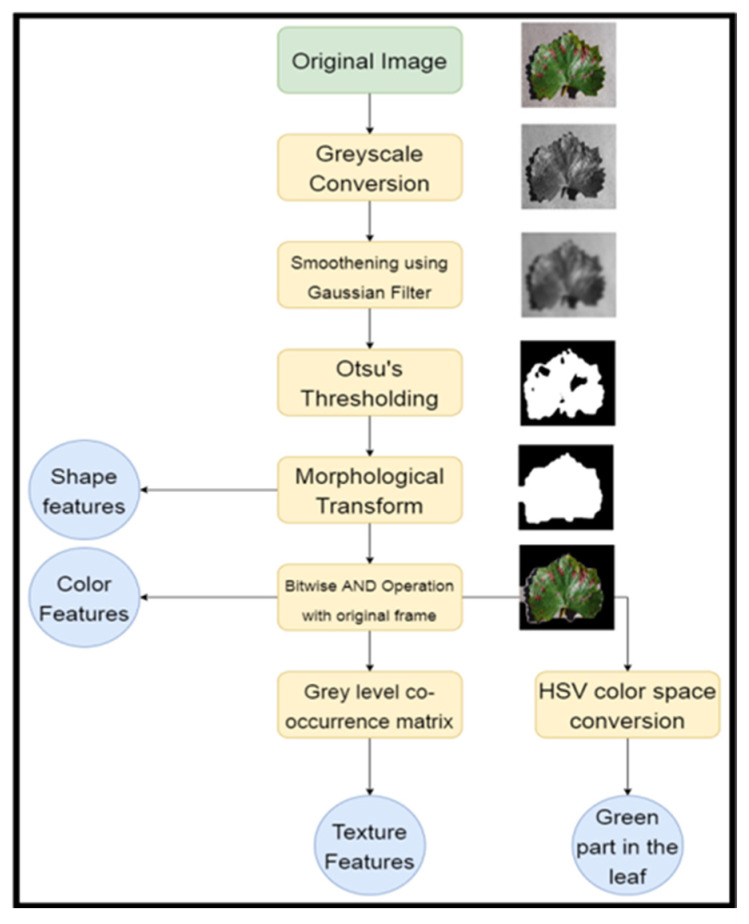
Basic data preprocessing techniques [16].

Figure 5 presents the preprocessing, feature extraction and classification pipeline employed in a basic plant disease detection model. The flowchart systematically outlines each step from the initial image acquisition to the extraction of texture, shape, and colour features essential for classification and analysis.

**Figure 6:**
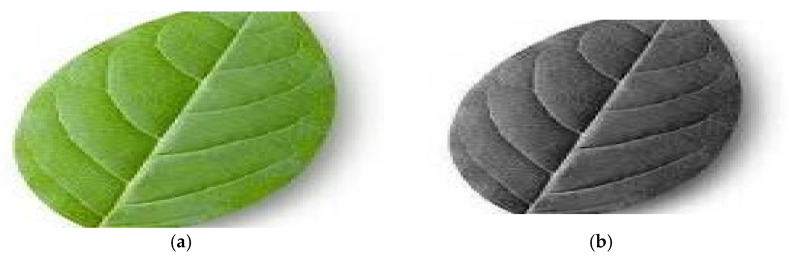
(**a**) Colour image; (**b**) grayscale image.

Acquired images are in colour (red, green and blue channels). They are often converted to grayscale during preprocessing to reduce computational complexity and to focus on shape/texture features. This technique converts colours to grayscale while maintaining differences between light and dark areas and keeping the level of brightness consistent [17]. Figure 6 displays the phenomenon, as Figure 6a shows the colour image before grayscale conversion and Figure 6b shows the results of grayscale conversion.

The RGB colour channels ($C_{rgb}$) have values that range from 0 to 1 based on the intensity of each colour in the image [17]. The values are calculated using the gamma expansion equation to make them linear, as shown by $C_{linear}$. Equations (6) and (7) ensure that the brightness of each pixel is represented accurately on a linear scale to ensure that no features are misinterpreted in the feature extraction stage.

$$\mathrm{If}\;C_{rgb}\leq 0.04045$$

(6) $$C_{linear}=\frac{C_{rgb}}{12.92}$$

Else

(7) $$C_{linear}=\frac{\left(C_{srgb}+0.065\right)}{1.065}$$

The grayscale luminance represented by $f\left(x\right)$ in Equation (8) displays the reflection of how the human eye sees the image [17]. The brightness of each pixel when the image is converted to grayscale is determined by the weight of each of these channels, determining how grey each pixel in the image is to preserve the image accuracy. *R* is the weight of the red pixels, *G* is the weight of the green pixels, and *B* is weight of the blue pixels.

(8) $$f\left(x\right)=0.2989\times R+0.5870\times G+0.1140\times B$$

In a study of leaf image detection using colour and texture features, the grey values of the three-colour channels were normalised according to the intensity of each, allowing for effective transformation of the image to the HSV colour space for easy background segmentation [18]. The grayscale technique does not eradicate the noise in the image, which necessitates the need for image denoising to ensure enhanced detection [19]. One of the most used denoising techniques is the Gaussian filtering, as shown by Equation (9), which is applied to smooth the image and reduce noise to enhance the accuracy of subsequent thresholding operations. This technique is displayed in Figure 4.

(9) $$G_{smoothedImage}=\left(\frac{1}{2\times \pi \times \alpha^{2}}\right)\times e^{-\frac{\left(a^{2}+b^{2}\right)}{2\times \alpha^{2}}}$$

This filter handles the impulse noise along with smoothening or blurring the image using the standard deviation (α), whilst a and b are the distance of the pixel from the horizontal axis and vertical axis, respectively.

Building upon this preprocessing foundation, a study to develop a model for the detection of sorghum diseases was conducted. The researcher began by converting RGB images to grayscale. This conversion enhances contrast differentiation, which is essential for accurate edge detection. The Canny Edge Detection algorithm was then applied to identify the edges of infected and healthy regions in the leaf images [20]. The images displayed image noise such as pepper and salt, shot noise and Gaussian noise [20]. To further enhance the quality of the segmented images, thresholding was performed to smooth the image and reduce noise. For feature extraction and classification, the AlexNet algorithm was employed. AlexNet consists of five convolutional layers, which are responsible for learning spatial hierarchies of features, and three fully connected layers that perform classification based on the extracted features [20].

Table 2 tabulates the advantages and disadvantages of some preprocessing techniques that were explored in a study for image preprocessing of the fundus image dataset. It highlights the trade-offs of most preprocessing techniques, which are between noise reduction and preservation of critical features such as image textures and edges.

This also highlights the necessity for a thorough examination of filter performance and its effects on the model’s computational efficiency, especially in the context of plant diseases, where the feature structures influence the type of disease detected.

Following image processing, segmentation techniques are applied to categorise an image into discontinuity and similarity. It mainly looks at the sudden intensity changes and similarity properties, which group similar features such as texture, brightness and colour. The technique comprises three sub-techniques: region-based, edge-based, and hybrid techniques. Region-based focuses on similarities between neighbouring pixels, edge-based segmentation focuses on the discontinuities in the image pixels and the hybrid technique is a mixture of the region-based and edge-based techniques [22].

Among segmentation techniques, Otsu Thresholding is widely used for its simplicity and effectiveness in separating the foreground objects from the background. In a study of flower segmentation, this technique was employed to optimally segment the leaf foreground from the background by maximising the variance ($\sigma_{b}^{2}\left(t\right)$) between pixels [23]. This method determined a threshold value to binarise the image such that intra-class variance is minimised using Equation (10). The grayscale image is an input, which, after thresholding, results in a binary image where the leaf is distinctly separated from the background.

(10) $$\sigma_{b}^{2}\left(t\right)=\omega_{0}\left(t\right)\cdot \omega_{1}1\left(t\right)\cdot {\left[\mu_{0}\left(t\right)-\mu_{1}\left(t\right)\right]}^{2}$$

where:

- $\omega_{0}\left(t\right)$: Probability (weight) of class 0 (background) up to threshold *t*.
- $\omega_{1}\left(t\right)$: Probability (weight) of class 1 (foreground) from threshold *t* + 1 to end.
- $\mu_{0}\left(t\right)$: Mean intensity of class 0.
- $\mu_{1}\left(t\right):$ Mean intensity of class 1.

The binary image undergoes morphological transformations, which refine the segmentation by removing small artefacts and closing gaps within the object, as shown in Figure 5. The two commonly used morphological techniques are erosion and dilation. Erosion removes the small white noisy dots to disconnect the slightly joint artefacts, whilst dilation expands the foreground by filling the small holes and connecting the nearby white pixels in the image [24]. This step improves the accuracy of the extracted shape features, which are derived from the leaf’s contour and structure. In parallel, the morphologically transformed binary mask is subjected to a bitwise AND operation with the original colour image. This isolates the leaf from the background in the RGB space, allowing for the extraction of colour features.

In a study aimed to evaluate segmentation techniques in corn, potato and tomato images, the ROC-AUC and Support Vector Machine (SVM) algorithms were employed to assess the classification performance. The researcher performed segmentation to isolate the areas of interest, specifically the infected regions of the plant leaves [25]. The segmentation process involved the use of the K-means clustering algorithm and Otsu’s thresholding technique. Initially, RGB images were converted to the HSI (Hue, Saturation, Intensity) colour space to enhance feature visibility. Further segmentation was achieved using boundary and spot detection algorithms to identify disease-affected areas accurately. The results displayed that the K-means algorithm is more efficient than the Canny edge and KNN algorithms in potato and tomato images [25]. Whilst the Canny edge detection algorithm performed efficiently in segmenting corn diseases with an accuracy of 94%, which is 14% above tomato and 8% above potatoes [25].

Table 3 summarises commonly used image segmentation techniques, outlining their contributions and limitations in the context of plant disease detection. While methods such as K-means clustering and neural networks offer efficiency and accuracy, they also present challenges such as parameter tuning and training complexity. These trade-offs must be considered when selecting segmentation approaches for real-time plant disease identification.

Feature extraction contributes immensely to object detection by enabling automated identification and plant disease detection through feature recognition such as shape, edges, colour or texture [27]. This also serves as a basis for disease classification. In plant disease detection, these features are vital for distinguishing between healthy and infected leaves. Accurate detection relies on selecting the most informative features in the image and applying robust feature extraction techniques that can accurately capture the subtle variations caused by different infections [27].

In a study for disease detection of 20 diseases in 5 common plants, the Grey Level Co-occurrence Matrix (GLCM) was utilised for feature extraction. The isolated leaf region is passed through a GLCM to obtain texture features, which describe the spatial relationships of pixel intensities and help to differentiate between healthy and diseased areas based on surface patterns [16]. The image is also converted into the HSV colour space, facilitating the analysis of colour characteristics by separating chromatic content (hue and saturation) from the intensity value, achieving an accuracy rate of 93% [16]. This assists in identifying the green part of the leaf, which is particularly useful for detecting discolouration due to disease. Overall, the described process enhances the model’s ability to effectively classify plant diseases by ensuring that essential features such as shape, texture, and colour are accurately extracted and normalised before being input into the classification model.

Building on feature extraction techniques, researchers have explored various statistical and handcrafted features for disease detection. In one study, researchers employed a comprehensive set of 12 colour features, such as mean, standard deviation, skewness, and kurtosis, alongside shape descriptors derived from HVI moment invariants, and texture features extracted via Local Binary Patterns (LBP) and GLCM [28]. Using this multi-dimensional feature set, the study achieved a classification accuracy of 86.58% with the XGBoost algorithm and 81.67% with an SVM algorithm across three distinct rice diseases, demonstrating the effectiveness of advanced machine learning models in a natural agricultural environment [28].

In the context of disease detection, feature extractors serve as an essential tool for dimensionality reduction, effectively projecting high-dimensional data into lower-dimensional spaces. This process enhances classification accuracy and addresses the challenges posed by the curse of dimensionality [29]. This results in models that not only have improved computational efficiencies but also have high model generalisation and interpretability.

Table 4 provides a detailed overview of various feature extraction techniques, emphasising their roles in image processing, particularly within the realm of plant disease detection. Additionally, it outlines the limitations associated with each method, thereby facilitating a comprehensive understanding of their applications and constraints.

To summarise, efficient preprocessing, segmentation, and strong feature extraction combined with deep learning create a synergistic pipeline that greatly improves the accuracy and reliability of plant disease detection models in practical agricultural environments.

## 3. Machine Learning in Plant Disease Detection

### 3.1. Overview of ML Approaches in Agriculture

**Figure 7:**
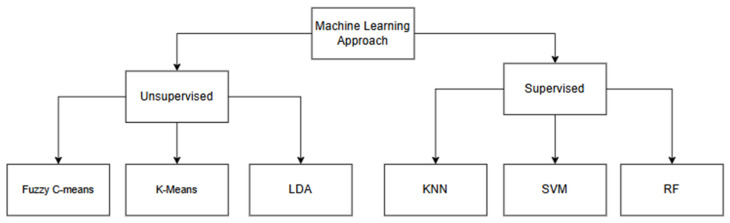
Categorised ML approaches.

ML techniques were among the earliest AI-based approaches applied to plant disease detection. Classical ML methods such as SVM, kNN, Random Forest (RF), and decision tree have shown promising results in the classification of diseases based on handcrafted features extracted from plant images. These features typically include texture, colour histograms, shape descriptors, and statistical measures. ML is categorised into supervised and unsupervised learning. Supervised learning requires labelled image datasets, where the disease type or healthy status is known, enabling the model to learn from input-output pairs [30]. On the other hand, unsupervised learning uses unlabelled image datasets and seeks to uncover hidden patterns or groupings within the dataset without prior knowledge of disease labels. The performance of these models depends on the quantity and quality of the training samples and can also be affected by the type of ML algorithm used [30]. Figure 7 illustrates the various types of ML algorithms that can be adopted for plant leaf disease detection.

#### 3.1.1. Support Vector Machines (SVM)

**Figure 8:**
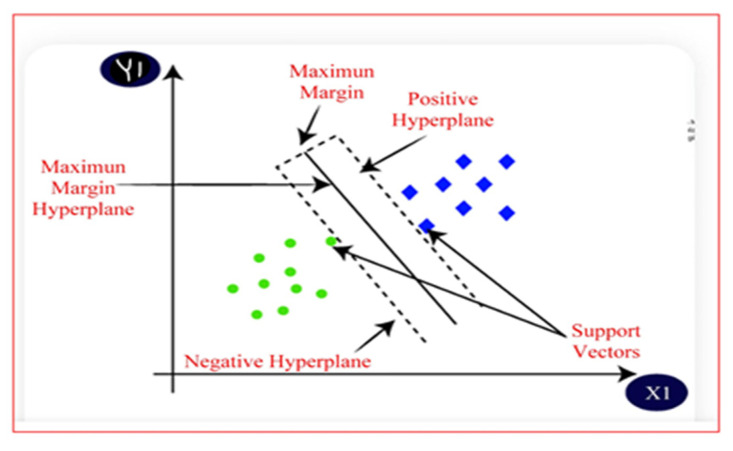
An illustration of the SVM algorithm in 2D [31].

Support Vector Machines (SVMs) are supervised learning models that utilise associated algorithms to perform both classification and regression tasks [31]. The main aim of an SVM algorithm is to find the optimal maximum margin hyperplane in n-dimensional classification, as displayed by Figure 8. Their performance is highly effective when the dataset under test has highly discriminative features [32].

Similar feature data are grouped to form clusters, enabling the SVM to distinguish between categories effectively.

Figure 8 illustrates how SVM separates two classes using an optimal hyperplane. Given training data $\left(x_{i},y_{i}\right)$ where $y_{i}\in \left\{-1,+1\right\}$, SVM finds the best hyperplane defined by the equation v⋅x+b=0 that separates the two classes. v represents the weight vector that determines the orientation of the hyperplane, and b is the offset that shifts the hyperplane from the origin. SVM ensures correct classification using Equation (11), where $x_{i}$ is the input feature vector for the i-th training image, and $y_{i}$ represents its corresponding class label [33].

(11) $$y_{i}\left(v\cdot x_{i}+b\right)\geq 1\;i\;=\;1,2\ldots \ldots ,\;N$$

The data points that lie exactly on the margin boundaries are called support vectors, and they satisfy Equation (12).

(12) $$y_{i}\left(v\cdot x_{i}+b\right)=1\;or=-1$$

These margin boundaries are given by the equations for the positive class, Equations (13) and (14), for the negative class.

(13) v⋅x+b=+1

(14) v⋅x+b=−1

The distance between these two hyperplanes is called the margin, and it is equal to $\frac{2}{\left|\left|v\right|\right|}$. SVM aims to maximise this margin while ensuring that all points are correctly classified [33].

#### 3.1.2. K-Nearest Neighbour (kNN)

**Figure 9:**
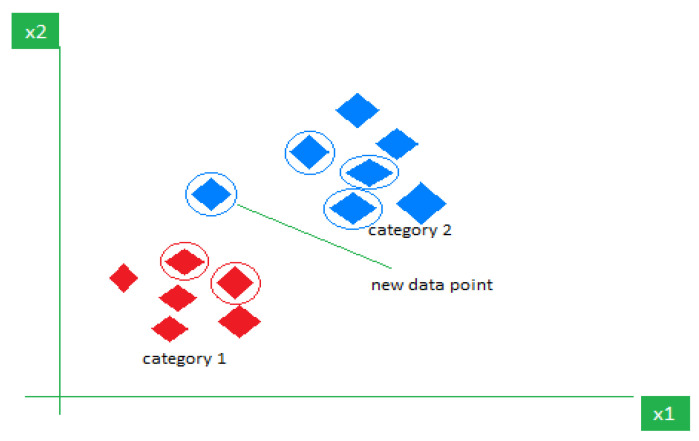
kNN algorithm working visualisation [35].

The kNN algorithm is a supervised, non-parametric algorithm that is used for classification based on feature similarity. The method compares new samples to the k-nearest stored samples and classifies based on majority voting, as displayed in Figure 9. Despite its high accuracy in controlled environments (e.g., 99.96% in certain studies), it is computationally intensive and may not scale efficiently with larger datasets [34].

#### 3.1.3. Decision Tree (DT)

DT is a supervised learning model that is mainly used for classification and regression tasks. This technique works by recursively splitting the dataset based on input features to create a tree-like structure, where internal nodes represent decision rules, and leaf nodes represent outcomes. The quality of a split is often measured using Entropy and Information Gain (*IG*). Entropy in Equation (15) quantifies impurity in a dataset D and is defined as:

(15) $$H\left(D\right)=-\sum_{i=1}^{n}p_{i}{log}_{2}p_{i}$$

where $p_{i}$ is the probability of class i in the dataset. *IG* in Equation (16) measures the reduction in entropy after splitting on attribute A, where *Dv* denotes the subset of *D* for which attribute *A* has value *v*, and ∣*Dv*∣ is the number of instances in that subset.

(16) $$IG\left(D,\;A\right)=H\left(D\right)-\sum_{v\in Values\left(A\right)}\left(\frac{\left|Dv\right|}{\left|D\right|}\right)H\left(Dv\right)\;$$

Though DTs are simple to implement and interpret, they have a drawback of being highly prone to overfitting, especially in cases where the dataset is large or is noisy. This introduces a trade-off between the tree’s complexity and its generalisation ability. Techniques such as combining unpruned DTs (Random Forest), pruning have been introduced to mitigate this drawback.

#### 3.1.4. Random Forest (RF)

RF is an ensemble supervised learning method that constructs multiple decision trees and aggregates their results for final classification. RF models are particularly robust to overfitting and can handle both categorical and numerical data efficiently. The RF model can be formally expressed in Equation (17) as:

(17) $$h\left(x,Tk\right),\;\;\;\;\;\;k=1,2,\ldots ,L$$

Here, x represents the input data, and Tk denotes a mutually independent random vector parameter that guides the construction of each k-th decision tree. Although this methodology introduces a drawback of increasing the model’s bias and reducing its interpretability due to the complexity of combining multiple trees, it significantly enhances the model’s predictive performance and robustness. Random Forest is known for its versatility and ease of implementation. It performs well even with high-dimensional data and yields results at a relatively faster rate, making it a reliable classification method in plant disease detection models.

### 3.2. Performance Comparison

Table 5 provides a summary of several classical ML models applied in different plant disease detection studies, including the image dataset utilised, achieved accuracy, and associated limitations.

**Figure 10:**
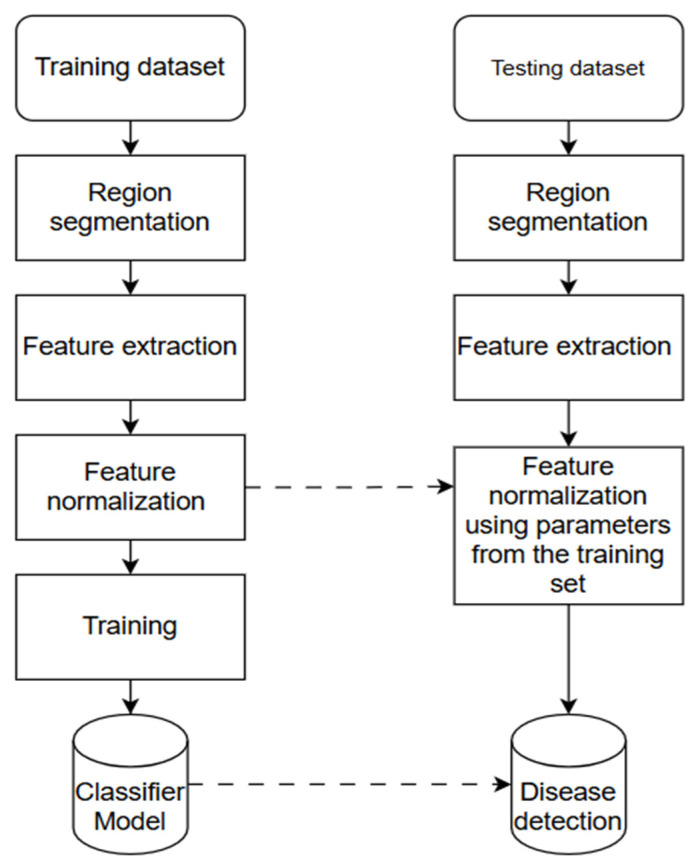
Classic training and testing phases for a machine learning model [41].

The ML model’s varying performance in Table 5 can be credited to the data preprocessing techniques, feature selection techniques, image dataset quality and size, and the model-specific limitations such as computational costs and hyperparameter tuning of each model. Though models such as kNN and DT achieved high performance accuracies compared to other ML models, that performance is limited to the controlled test dataset that was used in the testing stage. When deployed to real-time environments, these models are negatively influenced by environmental complexities, feature reliance and low model robustness due to inadequate generalisability. Illustrated by the kNN model, which achieved an accuracy of 99.96% on a PlantVillage dataset, which is a dataset benchmark that is collected under controlled lab conditions [38]. The general training and testing phases for ML models are illustrated in Figure 10.

The RF algorithm performed well in the admission prediction system developed by the researcher in their paper, where the RF was utilised as a classifier [42]. In a study in India to detect rice disease (bacterial leaf blight, leaf blast and brown spot) in their early growth development stages, ML techniques were utilised. Intensity Moments techniques were utilised to extract features from the image dataset, whilst RF was used to classify the images with infected plants from the healthy plants [43]. The dataset consisted of 352 images, which were used for training the model, whilst 176 images were used for testing. The methodology obtained an accuracy of 91.47% [43].

In another study of oil palm leaf disease detection, the Principal Component Analysis (PCA), a feature engineering technique, was used to reduce the model’s dimensionality and highlight critical features that differentiated the diseased leaves from healthy leaves. This technique was utilised to create 41 features, which were then processed by the L*a*b, RGB, HSV and HIS colour spaces to extract features by splitting the histogram of each channel [44]. K-means clustering, an unsupervised ML approach, was utilised to segment the region of interest (ROI). The results showed the proposed methodology produces good performance, which is displayed by the sensitivity, specificity, and accuracy rate that reached 99.3%, 100%, and 99.67%, respectively [44].

For tomato leaf disease detection, researchers resized the input image dataset to 256 × 256 pixels as one of the preprocessing techniques and coupled it with a histogram equaliser to enhance the image quality [45]. The leaf boundaries are extracted through contour tracing, and feature extraction methods such as Discrete Wavelet Transform (DWT), PCA, and GLCM are employed to capture significant characteristics of the leaf samples. The model then employs SVM, CNN and kNN for classification, which acquired 88%, 97% and 99.6%, respectively, on the detection of disordered tomato leaf images [45].

To combat the labour-intensive and fast spread of rice diseases, Bangladeshi researchers designed an automated system that used preprocessing techniques for background cancellation and trained the models using kNN, decision tree, Naïve Bayes and Logistic Regression (LR) using the same training dataset [46]. The models were tested using the same test dataset. The results of the decision tree model had an accuracy rate of 97.9167%, which was better than that of the LR, Naïve Bayes and kNN, which had 70.83%, 50% and 91.66%, respectively [46].

**Figure 11:**
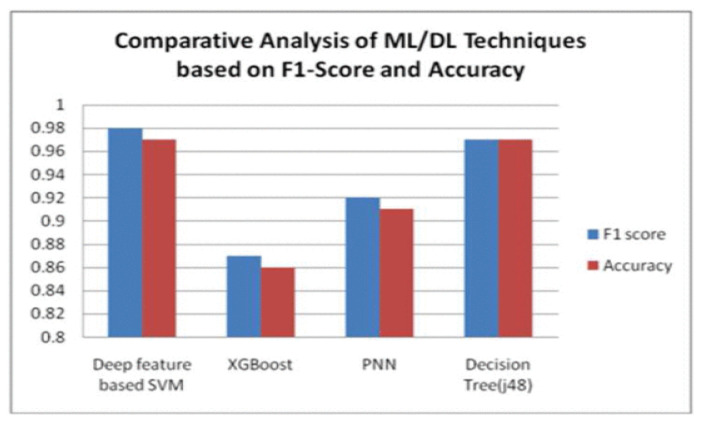
Classification report for testing fungal disease in rice images using ML algorithms [47].

To revolutionise rice from fungal diseases, wavelet analysis and PCA were used for feature extraction due to their efficacy in retaining feature information, which is vital for classification. The SVM classification technique was utilised as a classifier for its ability to handle complex data that may possess non-linearity [47]. The results of the model evaluation in Figure 11 show that the SVM-based model was efficient and that the XGBoost, PNN and decision tree algorithms as their F1 score and accuracy rates were higher [47].

The SVM model consistently outperformed others across key metrics such as F1 score and accuracy. This underscores the effectiveness of SVM in handling complex classification tasks in plant disease. While models like decision tree and XGBoost also showed competitive performance, the SVM’s robustness and ability to capture non-linear patterns in the feature space highlight its utility for high-stakes agricultural applications [47].

In real-world agricultural environments, the models may struggle due to increased data variability and environmental complexity, which includes varying lighting conditions, overlapping leaves. As classical ML models are trained for controlled lab conditions, with fixed, handcrafted features, they struggle to generalise in real-world conditions, thus making them unsuitable for real-world conditions, regardless of their high accuracy or robustness. These findings highlight the need for models that can be utilised in controlled and real-life agricultural environments. It also underscores the need for the adoption of deep learning, which will automate feature extraction and improve robustness across varying datasets.

The model architectures used by researchers in [42,43] are similar to the classifier model shown in Figure 9, where the focus is on image data preprocessing, segmentation, and feature extraction to improve the model’s robustness. In real-world settings, data augmentation, feature selection optimisation, and noise robustness are essential. ML models lack the flexibility of deep learning models like CNNs and ViTs, which can automatically extract features at multiple scales and better handle noisy inputs. Therefore, models should be evaluated not only on laboratory accuracy but also on their resilience to domain shifts caused by deployment environments.

### 3.3. Limitations of ML Algorithms

The reliance on handcrafted features can introduce bias and may not generalise well to complex or overlapping disease symptoms [48]. Secondly, ML-based models often underperform in real-world scenarios where image quality varies due to lighting, occlusion, or background clutter [49]. ML approaches require careful feature selection and preprocessing, which increases development time and computational complexity. In practice, SVMs are known for their ability to handle high-dimensional feature spaces and produce high classification accuracy. However, the performance of ML models largely depends on the quality of the extracted features, which requires domain expertise and often limits scalability across various plant types and environmental conditions [50].

Classical ML techniques have laid essential groundwork in automated plant disease detection, particularly through interpretable models based on handcrafted features. However, their limitations in scalability and generalisation display the need for deeper integration of advanced deep learning methods, which can autonomously extract robust features and adapt across diverse agricultural environments. Future research should aim to combine the interpretability of classical methods with the automation and precision of modern deep learning architectures.

## 4. Deep Learning Models

The introduction of Deep Learning (DL), a subset of ML, has improved the research on early disease detection and classification in plants, as the model accuracy levels have increased [51]. DL techniques extract high-level features and represent them in layers that follow a hierarchical structure [52]. Each of those features has been proven to be highly effective in image detection and classification [52]. The techniques have been utilised to enhance farming and improve crop management practices, leading to fewer losses due to disease infections.

### 4.1. Convolutional Neural Networks (CNNs)

**Figure 12:**
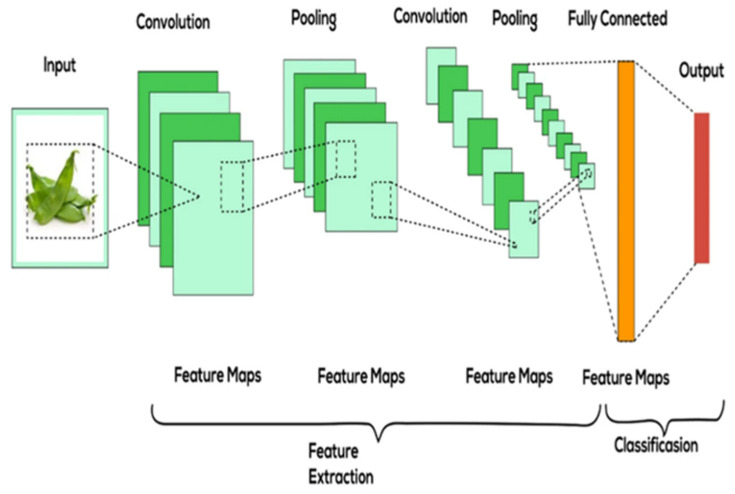
Traditional CNN architecture [53].

Deep learning has transformed plant disease detection by removing the need for manual feature engineering. CNNs are a subset of DL, excels in image classification tasks because they can automatically learn spatial hierarchies and intricate patterns from raw image data using the model’s multiple layers [52]. Figure 12 displays the model architecture of the CNN model, highlighting the hierarchical importance of each layer for feature extraction and classification [53].

The convolutional layer comprises convolutional filters that convolve the input image, represented in n-dimensional metrics, to produce an output feature map, as illustrated in Figure 12 [51]. The pooling layer, functioning as a sub-sampling stage for the feature maps, reduces their size while maintaining the essential features of the image [51]. The fully connected layer functions as a basic classifier; it is connected to the end of the architecture with neurons [52]. It utilises learned features to make decisions, such as identifying the type of plant disease in an image. In a neural network, an activation function is applied to introduce non-linearity to the model, thereby limiting the chances of overfitting or underfitting with the test dataset [52].

CNNs such as AlexNet, VGGNet, and ResNet have been utilised in various studies to classify plant diseases with impressive accuracy [54]. These models comprise convolutional layers for feature extraction, pooling layers for dimensionality reduction, and fully connected layers for classification [54]. Although these techniques are adopted by CNN architecture, they differ from one another. The AlexNet model consists of 5 convolutional layers coupled with 3 fully connected layers that use the ReLU activation [55]. The ReLU function, as displayed in Equation (18), reduces the computational load during modelling [55]. Additionally, it uses max-pooling layers to down-sample the spatial dimensions of the feature maps [56].

(18) $$ReLU\left(x\right)=\max\left(0,x\right)$$

VGGNet is often modelled using 16 or 19 layers [57]. When compared to traditional CNN architecture, it represents an improved version, as it replaces large filters with smaller 3 × 3 convolutional layers that enable the model to extract deeper features [57]. Similarly to the AlexNet algorithm, it utilises max pooling for down-sampling the spatial size [56]. The ResNet technique introduces residual connections that address the vanishing gradient issue in deep networks. It employs residual blocks where the input of a layer is added to the output of the subsequent layer, allowing it to extract more features in a relatively shorter time frame [58]. ResNet models have shown better performance compared to other well-known convolutional neural network (CNN) architectures like VGG16 and DenseNet [58].

Table 6 illustrates the performance of various CNN-based models and how those different architectures have been successfully applied by researchers to plant disease detection tasks across multiple datasets, crops, and disease types.

When comparing the accuracy rate of models in CNN-based models in Table 5 with ML-based models in Table 6, it is evident that CNN-based models outperform ML-based models in image recognition and processing tasks. This superiority is due to the core capability of CNNs to automatically learn hierarchical features directly from raw image data. This makes them more architecturally complex compared to traditional ML models, thereby eliminating the need for manual feature selection and engineering, which is typically required in most traditional ML algorithms. This is mainly due to their convolutional and pooling layers, which allow CNNs to extract spatial and structural patterns from images with minimal preprocessing, resulting in improved accuracy, particularly in disease classification tasks where visual patterns may be subtle or complex.

However, their performance tends to degrade in real-world applications due to the presence of occlusions, poor lighting, variable camera angles, and complex backgrounds that are rarely seen in training data. This environmental variability directly impacts the model’s ability to generalise, leading to increased misclassifications in the field.

The architecture and complexity of CNN algorithms also affect the processing or detection time in a single-image acquisition. The more complex the model, offer better performance on training data due to their ability to capture the smallest features. But this is also a trade-off as the model may also require a higher processing time, which hinders real-time classification.

In a disease detection study, a dataset comprising a combination of real-world images and publicly available data was used to develop a model for identifying infected plant images. The researchers developed several CNN-based models, which were compared to determine the most suitable option for real-time deployment.

Table 7 illustrates how AlexNetOWTBn, an improved version of AlexNet through parameter tuning and batch normalisation. Thus, this model achieved a higher accuracy than more architecturally complex models such as GoogleNet, which uses inception modules, and VGG, which contains significantly more parameters.

While VGG achieved 98.87% accuracy in a controlled test environment, it required more than 4000 s per epoch, which makes it an unsuitable model for real-time applications where high inference speed is critical. Conversely, lightweight architectures like MobileNet and YOLOv3, although slightly less accurate in ideal settings, offer better inference speed and computational efficiency, making them more practical for field deployment. YOLOv3, for instance, balances real-time object localisation and classification with minimal delay, even under variable conditions.

**Figure 13:**
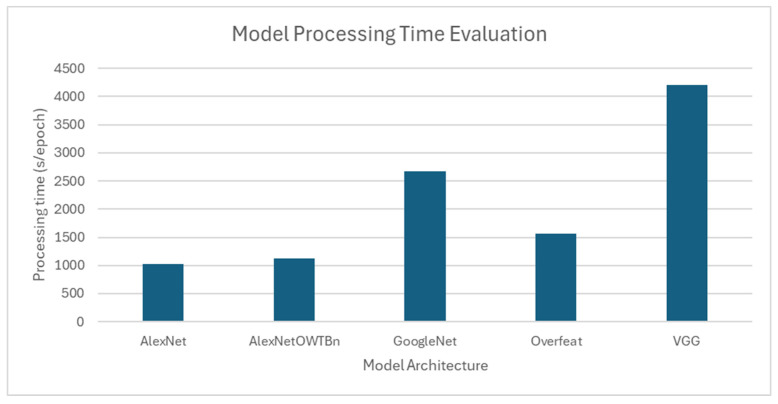
Processing time comparison of various CNN-based models for the same dataset.

Figure 13 also highlights how model complexity impacts processing time; for instance, VGG requires substantially more time per epoch than AlexNetOWTBn. Given its high computational requirements, the VGG architecture may be more appropriate for laboratory-based environments rather than real-time field applications. Although high-complexity CNNs may suit precision analysis in lab-based research or post-harvest inspection, lightweight CNNs or YOLO-based architectures are better aligned with the demands of on-field, real-time, and mobile plant disease detection.

### 4.2. Custom CNN Models for Specific Crops

To address crop-specific challenges, researchers have developed custom CNN architectures tailored to specific plant species. These hybrid CNN models are combined with traditional ML models for better performance [65]. Some have been used to detect early blight in potatoes, powdery mildew in grapes, and bacterial blight in rice. These custom models incorporate domain-specific knowledge and often combine CNNs with techniques such as transfer learning, data augmentation, and ensemble learning to improve robustness and generalisability [65]. One of the most used architectures for customised plant disease detection is the You Only Look Once (YOLO) technique. This technique is best known for its inference speed and accuracy in the detection of plant diseases [66].

**Figure 14:**
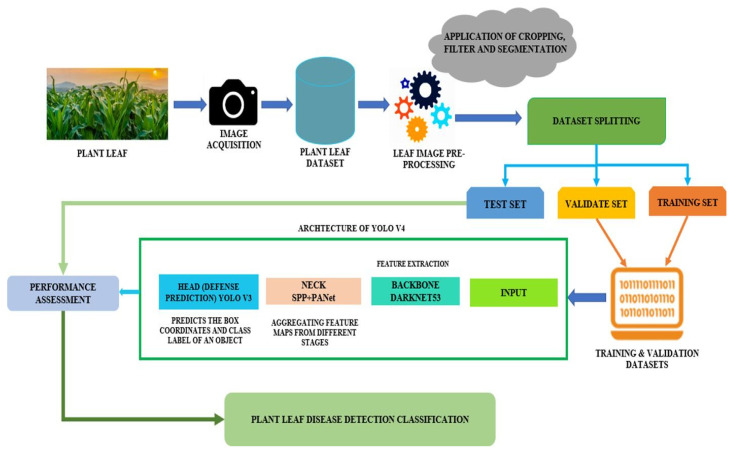
The YOLOv4 framework for detecting and identifying plant diseases [67].

Figure 14 displays the model architecture of YOLOv3 adopted by researchers in the investigation of improving plant disease detection in natural environments [67]. The model performs basic preprocessing techniques, image cropping to ensure that the input images are uniform, followed by noise filtering and segmentation to allow efficient feature extraction with no occlusions or unwanted background artefacts [67]. The architecture includes a backbone (DarkNet53) for model feature extraction, neck (spatial pyramid pooling (SPP) + PANet) where these algorithms are integrated for feature aggregation and enhancement, and head (YOLOv3) for predicting bounding boxes and class labels, classifying the boxes [67].

**Figure 15:**
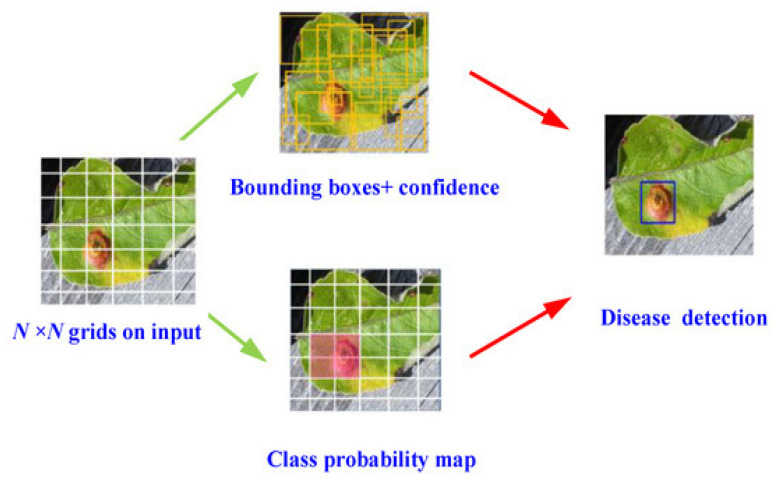
YOLOv4 schematic for plant disease detection [68].

The model processes leaf images by dividing them into grids, generating bounding boxes with confidence scores, and producing class probability maps, ultimately identifying disease-affected areas with high precision, as illustrated in Figure 15 [67,68].

**Figure 16:**
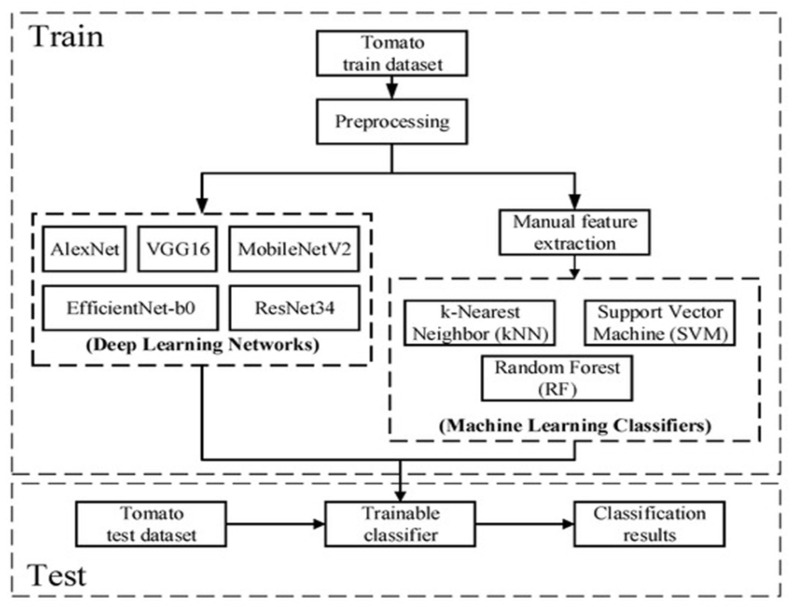
Flowchart of disease detection model using DL and ML techniques [69].

An experiment was conducted to compare classical machine learning (ML) and deep learning (DL) by testing tomato diseases from the PlantVillage dataset. Figure 16 shows the model architecture, particularly emphasising the CNN-based models utilised in this study. For classification purposes, the tomato dataset underwent preprocessing, which involved manually extracting disease features [69]. In contrast, the deep learning classifier, as one of the feature extractors, GLCM could automatically extract features, eliminating the need for manual extraction [67]. The pre-processed images and extracted features were fed into the DL and ML networks for training. Once the training was complete, they obtained the trained models, which were then used to classify the test dataset.

**Figure 17:**
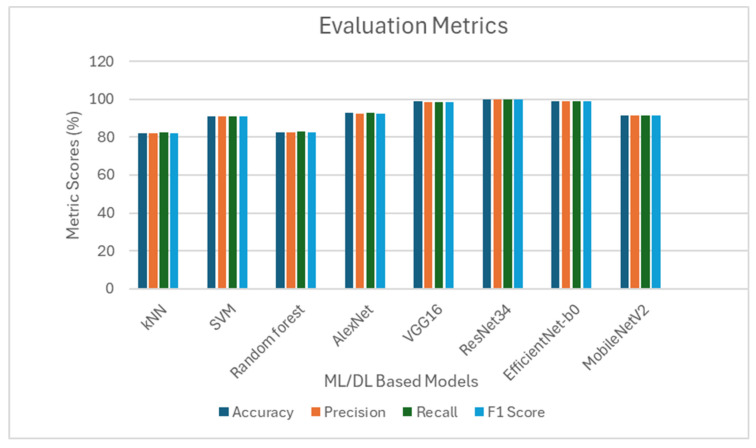
Results for the tested ML/DL algorithms [69].

The results from the accuracy, precision, recall, and F1 score evaluations, displayed in Figure 17, show that DL-based models performed better than ML models. Illustrating that sufficient feature extraction enhances the efficiency of DL models.

Classical ML models such as kNN and SVM, illustrated in Figure 16, are often highly accurate on structured lab datasets. Some exceed 99% accuracy, but they depend heavily on handcrafted features and suffer when exposed to image distortions, scale variations, or novel visual patterns not seen during training. This limits their scalability and adaptation to diverse agricultural scenarios.

Conventional CNN algorithms, such as those displayed in Figure 16, have demonstrated high accuracy and efficiency in controlled environments; their performance tends to deteriorate when used in real-time environments. This is mainly due to the challenges such as variable lighting, occlusions, background noise and limited computational resources. As a result, the number of misclassifications tends to increase under field conditions.

These limitations underscore the pressing need for developing lightweight, robust algorithms capable of delivering accurate and reliable plant disease detection in real-time agricultural settings. The introduction of algorithms such as the single-stage and two-stage aimed to address these drawbacks.

#### 4.2.1. Single-Stage Algorithms

A single-stage algorithm simultaneously performs classification and localisation of plant disease targets by directly extracting features from the network to predict both the disease category and its location [70]. These models are faster and more computationally efficient than conventional CNN algorithms, making them suitable for real-time disease detection. YOLO, Single Short MultiBox Detector(SSD) and RetinaNet algorithms are the most used single-stage algorithms due to their ability to extract features in a single network and be able to localise and classify targets in that network [70].

The YOLOv3 architecture focuses on basic preprocessing applications such as image cropping, noise filtering and image segmentation to standardise the input images and reduce the impact of occlusions and background artefacts. In modelling this algorithm, a simple structure of backbone, neck and head is used as discussed for Figure 13. In a study to detect early onset bacterial spot in bell pepper, YOLOv5, an improved YOLO algorithm, was utilised with an aim of using the bounding and anchor boxes to detect the smallest spot in the leaf at high speed and accuracy [66]. The final testing was implemented in a natural agricultural environment using a mobile phone and compared to other models developed with the same dataset and tested in the same environment. The confidence score was about 98.7% for YOLOv5, which is higher than 80% for SSD and 97.4% for YOLOv3, which struggles a bit with detecting small features [66].

The Single-Shot MultiBox Detector (SSD) integrates the one-stage regression approach of the YOLO series with the anchor box mechanism from Faster R-CNN. It utilises VGGNet as its backbone for feature extraction and generates detection predictions from multiple feature maps at different resolutions, ranging from shallow to deep layers [71]. This multi-scale strategy enables SSD to effectively detect both small-scale and large-scale objects. A key advantage of SSD lies in its ability to significantly enhance inference speed while maintaining high detection accuracy, making it well-suited for real-time applications.

When single-stage algorithms like RetinaNet and SSD were evaluated using diverse performance metrics, both models demonstrated high effectiveness. In a classification study involving cats and dogs, the mean average precision (mAP) values were 89.1% for RetinaNet and 87.5% for SSD, indicating strong performance across the board [72]. RetinaNet particularly excelled in managing class imbalance, while SSD demonstrated its adaptability and suitability for real-time applications due to its high inference speed [72].

Although these results were promising for advancing object classification, they also revealed a fundamental trade-off between precision and speed in single-stage algorithms. RetinaNet prioritised accuracy through complex computations, whereas SSD emphasised speed, which may come at the cost of slightly reduced precision.

In an attempt to investigate accurate feature extraction across a Kaggle-adopted dataset featuring 14 plants, a deep block attention SSD model was compared to the squeeze-and-excitation SSD (Se_SSD) and deep block SSD (DB_SSD). Se_SSD focuses on feature extraction and the attention mechanism, while DB_SSD concentrates on feature extraction using the VGG network and the channel mechanism. The accuracy rate (mAP) of the DB_SSD at 92.20%, demonstrating the performance boost offered by the deep block attention module [71].

In the context of single-stage algorithms, Table 8 discusses the strengths and weaknesses of the most commonly used techniques. The focus is to highlight the functionality of each algorithm in terms of its speed, accuracy and the trade-offs of each of these techniques. This comparison serves as a guide for selecting appropriate models based on specific application requirements, such as real-time detection needs versus high-precision classification in plant disease detection.

#### 4.2.2. Two-Stage Algorithms

Unlike single-stage detectors, two-stage algorithms such as Faster R-CNN or Mask R-CNN first generate region proposals, where preliminary tests are performed, where all positive samples are screened out, and ROI are generated before classification and bounding box regression is implemented [73]. These models generally achieve higher accuracy than one-stage algorithms, particularly for small or overlapping objects, though they come at the cost of increased computation and latency. These models are also limited to offline applications and have a high inference time [12]. Common two-stage object detection algorithms include Faster R-CNN, Region-based Fully Convolutional Networks (R-FCN), and Feature Pyramid Networks (FPN), among others.

**Figure 18:**
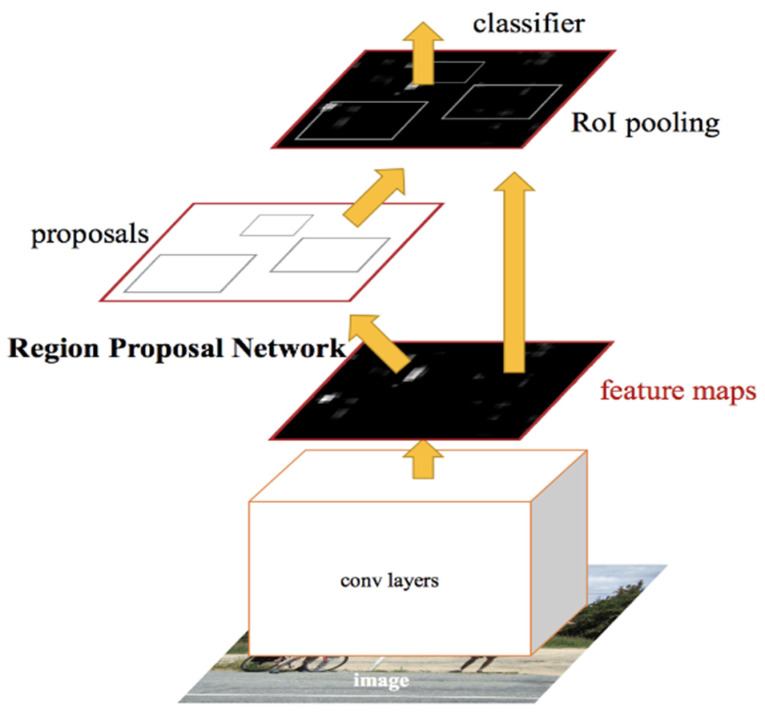
Network structure diagram of Faster R-CNN, which is an integration of RPN and Fast R-CNN [73].

The image passes through several layers for classification in Faster R-CNN, as displayed by Figure 18. First, the convolutional layer is used for feature extraction, followed by the Region Proposal Network (RPN), which takes the feature maps and generates potential object regions using anchor boxes. This acts as a segmentation stage, as the anchor boxes classify regions as either foreground or background. Region of Interest (RoI) pooling is then employed to resize the boxes to fixed dimensions, ensuring consistency in how the regions are processed by the network before classification.

There is an architectural difference between the R-FCN algorithm and Faster R-CNN, even though they are both two-stage object detection algorithms. R-FCN uses a fully convolutional network as its backbone, meaning that feature extraction and classification occur in the same convolutional layers. This eliminates the need for fully connected layers, making it more computationally efficient.

In an experiment on object detection using the two-stage technique, a dataset of 256 training images and 98 testing images was used to compare the four two-stage detector-based models, namely Faster R-CNN, R-FCN, FPN and Cascade R-CNN. The results displayed that integrating basic ML techniques in an R-CNN model improves its performance by 1.69% [73]. This is deduced from the Cascade R-CNN model, which had an addition of the HOG algorithm and uses different thresholds for object segmentation [73].

In the experiment of tomato plant disease detection, a model known as the Real-Time Faster Region Convolutional Neural Network (RTF-RCNN) was proposed, leveraging both static images and real-time video streams. The initially developed Faster CNN model had 12 layers, but it had a drawback of overfitting [74]. The researchers then proposed a 9-layer augmented R-CNN model to mitigate overfitting and improve model performance. The model’s performance was evaluated using metrics such as precision, accuracy, and recall, and compared against standard CNN architectures, including AlexNet and a conventional CNN model. The results demonstrated that the RTF-RCNN achieved an accuracy of 97.42%, outperforming AlexNet (96.32%) and the baseline CNN model (92.21%) [74].

The RTF-RCNN model outperformed the baseline CNN and AlexNet because it was designed to address overfitting by reducing the number of layers from 12 to 9, which helped improve the model’s generalisation without compromising its feature extraction. Unlike traditional CNNs, this model integrates a region proposal network (RPN), allowing it to focus on disease-affected areas of the leaf rather than the whole image, making classification more precise. It was also built to handle both static images and real-time video streams, which makes it more suitable for real-time field deployment. Since it was tailored for tomato leaf disease detection, the architecture could extract task-specific features better than general-purpose models. Its performance was further validated across precision, recall, and accuracy metrics, showing why it was more effective than standard CNN models for this task.

The literature discussed in this sub-topic highlights how researchers have used basic ML/DL techniques and integrated them with two-stage algorithms to address various drawbacks, such as speed and precision. Concluding the model comparisons on why some models performed better compared to others, even though they were deployed to the same deployment medium in the same conditions.

### 4.3. Limitations of DL Algorithms

While DL methods, particularly CNNs, have significantly advanced the detection of plant diseases, several limitations have been acknowledged by various researchers.

DL models such as CNNs heavily rely on large, high-quality annotated datasets. This poses a challenge in natural environments, as they mostly have uneven lighting contrasts with laboratories where the light source can be controlled to ensure accurate detection. Consequently, the effectiveness of models designed with lab-based datasets is limited in varied environments; the model’s accuracy diminishes in the presence of occlusions and background artefacts in the plant images. Training deep architectures like ResNet, VGGNet, or YOLOv4 requires substantial computational resources, including GPUs and memory. This demand can be impractical and costly for numerous field applications, particularly in developing regions with restricted technological access infrastructure.

Literature also highlights that even though CNNs can learn features automatically, preprocessing remains crucial for achieving high accuracy, which contradicts the aims of an end-to-end deep learning model that seeks to employ complex techniques. This suggests that there may always be a dependency on traditional image processing and future DL/ML techniques. Techniques such as YOLOv4 are designed for real-time detection; however, when deployed in uncontrolled environments (with occlusion and multiple leaves in the frame), the inference speed and accuracy can degrade significantly. This also raises the question of how traditional DL/ML techniques can be utilised to enhance the efficiency of real-time models in a natural environment.

## 5. Vision Transformers for Plant Disease Detection

**Figure 19:**
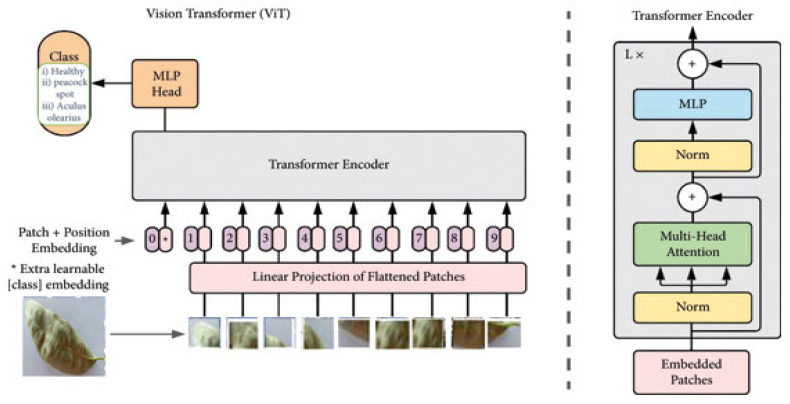
Basic Vision Transformer architecture for plant disease detection [76].

Vision Transformers (ViTs) are adopted from Natural Language Processing (NLP), where words/text are tokenised and processed as a sequence of embeddings for text recognition. However, in plant disease detection, an image is divided into a sequence of patches for image recognition. Figure 19 illustrates the basic architecture of ViTs, which includes a patch embedding stage that divides the image into patches, flattens each patch, and linearly embeds them into vectors [75]. Positional encodings are then added to retain spatial information. These patch embeddings are passed into the Transformer encoder, which uses multi-head self-attention, feed-forward networks, and layer normalisation to extract deep features [75,76]. A special classification token is appended to the sequence and is used to produce the final class prediction.

In recent years, ViTs have emerged as a competitive alternative to CNNs for image classification and object detection tasks. The algorithms’ ability to apply a self-attention mechanism across image patches allows the model to learn the global contextual features more accurately than CNNs, which mainly focus on features from the local receptive field [76].

### 5.1. Applicability in Plant Disease Detection

Accurate and efficient plant disease detection requires the model to identify subtle morphological, colour and texture deviations between the plant images in varying backgrounds and lighting conditions. CNNs have demonstrated strong capabilities when used for such modelling; however, they still face drawbacks with occlusions and background artefacts, which leads to researchers implementing various image processing techniques to ensure that there are fewer inaccuracies. While those techniques are effective, they mostly work in controlled environments and rely on specialised equipment. This makes it impractical to deploy such models in real-time, as it would be expensive to acquire some of the equipment. CNNs do not consider the position of each pixel and the relationship it has with pixels in the image. Researchers have thus adopted ViTs due to their capacity to model long-range dependencies and handle large datasets efficiently.

ViT modelling was utilised in a study to identify healthy tomato leaves from infected tomato leaves, detecting 10 varying diseases. The model dataset consisted of 10,010 images, and the model was compared to a CNN-based Inception-V3 model [77]. The validation accuracy results were 95.76% and 88% for ViTs and Inception-V3, respectively [77]. The performance of these detection techniques is reliant on the dataset size and the preprocessing techniques for modelling. In this case, Inception-V3 performed less than the ViT model because it is sensitive to spatial hierarchies and is limited to the receptive field, especially as it was faced with a high intra-class variability present in the model dataset.

In an effort to enhance the Vision Transformer (ViT) architecture, several models have been proposed with a focus on leveraging its self-attention mechanism. The Swin Transformer, for example, introduced a shifted window technique that enables efficient computation of local attention while preserving cross-window connections. This approach contributes to improved scalability and accuracy of the model as the transformer uses hierarchical structures with patch merging, thus reducing spatial dimensions, allowing it to perform efficiently in high-resolution images [78]. This technique tends to perform better than traditional ViTs, which maintain a constant feature resolution.

The Swin Transformer was utilised to design a triple-branch algorithm on an AI Challenger dataset, where each of the three modules was used for feature extraction, severity classification and deep supervision. The model was benchmarked by 5 widely used classification networks, namely, ResNet34, ResNet50, VGG16, VGG19, traditional Swin Transformer and the proposed Triple-branched Swin Transformer. The Triple-branched Swin Transformer outperformed the other network in all severity classes, 99.60% for healthy, 81.95% general infected and 87.80% for serious infections [79].

The GreenViT algorithm was proposed for the detection of plant infections, as CNN-based models tend to exhibit dimensional information loss when applied to plant images with similar visual features [80]. GreenViT builds upon the strengths of traditional Vision Transformer (ViT) models, particularly their self-attention mechanism, which captures long-range dependencies across image patches, making them more effective in complex classification environments. By fine-tuning the ViT architecture and reducing the number of parameters from 86 million to 21.65 million, GreenViT achieves a smaller model size, resulting in improved image classification speed suitable for edge deployment [80].

All benchmark CNN-based models, the traditional ViT, and the GreenViT model were trained for 10 epochs using a low learning rate to retain the information learned in each epoch. Table 9 presents the inference speed of the benchmark models and the proposed GreenViT when deployed on an edge computing device and tested in a real-time environment. The CNN-based models (MobileNetV1, MobileNetV3Small, and EfficientNetB0) demonstrate higher inference speed (Frames Per Second, FPS) and lower model sizes compared to the ViT-based models. This advantage is primarily due to the efficient architectural design of CNNs, which exploits spatial locality and translation invariance through transfer learning, allowing them to process local features using fewer computational resources and less processing time per image.

However, CNNs do not explicitly consider the positional relationships between pixels in an image, which limits their effectiveness in dynamic real-world settings. This often results in reduced accuracy and robustness under varying illumination, occlusion, or background conditions. Consequently, CNN-based models are best suited for deployment in controlled environments with minimal variations in lighting and occlusion.

### 5.2. Multimodal and Self-Supervised Extensions

To improve the performance of ViTs in plant disease detection, researchers have developed models that incorporate multimodal learning and self-supervised learning (SSL) techniques. Multimodal learning involves acquiring data from multiple sources, such as hyperspectral imagery, RGB images, and thermal data, to provide a richer context for accurate disease detection. The integration of sensor information into the model enhances classification robustness, particularly in real-time environments.

In a study focused on grape disease detection in natural environments, a transformer-based multimodal framework was developed. The model integrated RGB images, hyperspectral data, and environmental sensor inputs to perform effectively under diverse conditions, including variations in lighting, humidity, and temperature. Although the model achieved an impressive accuracy of 94% during real-time deployment via a mobile application, it faced challenges related to computational limitations [81]. The initial framework required a large model size, which resulted in a high inference time, an undesirable trait for real-time applications.

To address this, researchers optimised the system by implementing a lightweight Transformer architecture with fewer parameters, thereby reducing the model’s computational overhead. Additionally, they incorporated a lesion-agriculture module, which generated interpretable, text-based disease reports to support decision-making by farmers in the field.

Despite these improvements, the model’s inference speed remained relatively slow at approximately 650 ms per image, which is still suboptimal for real-time use, especially in scenarios where rapid processing is essential. However, the inclusion of explainable outputs proved valuable, as it enabled farmers to better understand disease symptoms and facilitated more effective treatment administration.

A hybrid Vision Transformer–CNN (ViT–CNN) model trained on multispectral data from real-world plant disease images achieved an accuracy of 88.86% [82]. By leveraging the complementary strengths of Convolutional Neural Networks (CNNs) and Vision Transformers (ViTs), the model improved detection accuracy under field conditions. The dataset used was captured in a natural environment using lens filters and Near-Infrared (NIR) imaging, enhancing the model’s ability to detect subtle disease symptoms.

The hybrid architecture combined a ViT with ResNet50, a backbone known for its effectiveness in local feature extraction, while the ViT component contributed global self-attention capabilities. The model was evaluated under various configurations to assess how the number of parameters influenced inference speed and classification accuracy. Specifically, the ViT_r50_132 variant contained more parameters and required extensive feature engineering and fine-tuning compared to the ViT_r26_s32, which had fewer parameters. As a result, ViT_r50_132 underperformed due to its larger model size, which increased training complexity and made it more prone to overfitting, thereby reducing its testing accuracy.

Despite these challenges, the ViT–CNN hybrid model outperformed the benchmark DenseNet121, which achieved a lower accuracy of 76.69% [82]. These findings demonstrate that hybrid models offer significant advantages over traditional CNNs in natural environments. However, further research is needed to enhance their efficiency, scalability, and robustness for real-time agricultural applications.

SSL enables models to learn useful representations from unlabelled data through pretext tasks such as image inpainting and patch reordering in ViTs. This approach reduces the model’s reliance on annotated datasets, which are time-consuming and costly to produce. Since ViTs typically require large-scale datasets to perform well in classification tasks, SSL significantly enhances their scalability and applicability in data-scarce agricultural settings.

The study to develop a high-performance model for versatile plant disease detection involved fine-tuning a ViT-Large model, initially pretrained using Masked Autoencoding (MAE), on the PlantCLEF2022 dataset for real-time plant disease detection. The model’s average accuracy was 86.29%, considerably higher than the accuracy of the benchmark model, ResNet50, which achieved an average accuracy of 73.53% on the same dataset, comprising 12 plant diseases [83]. This self-supervised pretraining markedly improved downstream plant disease classification performance compared to models trained solely with supervised learning or traditional CNN-based methods. The results demonstrate that SSL-based models generalise disease detection more effectively than CNN benchmark models in natural environments.

To leverage unlabelled image data for plant disease detection, a self-supervised learning (SSL) model called Contrastive Vision Mamba was introduced. This model incorporates a Vision Mamba encoder, which captures long-range dependencies and enables the model to balance both local and global feature alignment effectively. The model was tested on 3 datasets, namely, PlantVillage, PlantDoc and the Citrus dataset, achieving accuracy results of 98.62%, 94.29% and 91.38%, respectively. These high accuracy rates stem from the model’s ability to extract rich semantic features from unlabelled data, improving generalisation and reliability when it is deployed in a real-world environment.

However, the variation in accuracy rates across the datasets can be attributed to differences in the resources and environments during the image acquisition stage. The PlantVillage dataset comprises images captured in controlled environments with minimal background noise and occlusions, using high-quality cameras under conditions that significantly enhance model performance. In contrast, the PlantDoc dataset includes images taken in natural environments, where occlusions, inconsistent lighting conditions, and complex backgrounds introduce noise and variability, slightly reducing accuracy. The Citrus dataset presents an additional challenge due to class imbalance, where some disease classes are overrepresented while others have relatively few samples. This imbalance negatively impacts the model’s ability to generalise across all classes, leading to a lower overall accuracy compared to the other datasets.

The model assessment was conducted against several state-of-the-art self-supervised learning (SSL) models, namely CAE integrated with CNN, CLA, CIKICS, and clustering-based models, all of which have demonstrated strong performance in diverse environmental conditions according to prior research [84]. The proposed ConMamba model achieved higher accuracy than all benchmark models, underscoring its superior capability in handling complex and variable agricultural settings. These results highlight ConMamba’s effectiveness in extracting robust features from unlabelled data and its potential for real-world plant disease detection applications.

### 5.3. Model Performance in Varying Conditions

The deployment of Vision Transformer (ViT)-based architectures for plant disease detection in real-world agricultural environments presents numerous challenges. These include varying lighting conditions, occlusions, background noise, weather fluctuations, sensor limitations, and differences in image acquisition techniques. Therefore, evaluating model performance under such diverse conditions is essential to assess their practical utility and robustness.

Unlike traditional Convolutional Neural Networks (CNNs), which are highly effective in controlled or lab-based environments, ViTs and hybrid ViT–CNN models demonstrate stronger resilience when deployed in unstructured and unpredictable field conditions. Their ability to model long-range dependencies and contextual relationships between image patches makes them well-suited to manage the inherent variability in real-world datasets.

Table 10 shows a comparison of various ViT-based and hybrid ViT models tested across different datasets and deployment settings. These models were assessed not only for accuracy but also for their robustness in real-time environments and their ability to generalise under real-time constraints where unwanted artefacts are present.

Lighting variations, background artefacts and occlusions are not the only environmental constraints that hinder CNN-based models from performing efficiently in real-world conditions. Humidity changes, plant posture variations and water droplets on camera lenses also form part of these constraints. The introduction of ViT architecture to plant disease detection assists in addressing these constraints through its ability to extract global features and consider pixel relationships within an image. This makes it easier for the model to adapt rapidly to real-world changes.

The integration of self-supervised learning (SSL) and multimodal learning significantly enhances model resilience and generalisation in diverse agricultural environments. Specifically, the application of SSL in plant disease detection addresses challenges such as class imbalance and limited dataset sizes, common issues in real-time image acquisition due to the labour-intensive and time-consuming nature of collecting labelled data. By learning meaningful representations from unlabelled data through pretext tasks, SSL enables models to leverage the full potential of available data without relying heavily on manual annotation, thereby improving classification performance in real-world environments.

Although the introduction of ViT addresses some of the environmental challenges, hardware limitations in the field also pose constraints in effective plant disease detection. Mobile devices and low-power IoT edge devices typically have limited computational power, memory and with the introduction of additional sensors for multimodal models, it becomes an added overhead in the detection algorithm. Thus, lighter ViT variants like Swin Transformer, GreenViT, or ViT hybrids with parameter tuning have been used due to their suitability for real-time applications. The inference speed, measured in FPS, becomes a critical metric. While traditional CNNs like MobileNetV1 show high FPS, they struggle in image diversity and generalisation, especially when no preprocessing is applied. Conversely, ViT-based models often achieve lower FPS but offer better robustness and adaptability.

## 6. Future Directions and Research Gaps

Having surveyed recent research papers in the domain of plant disease detection, the following research gaps have become apparent for progress in this research study:

- Datasets for African crops under natural conditions.
- Biases in datasets.
- Real-time models for disease severity grading, not just detection.
- Lack of standardised benchmarks.

The current research has demonstrated significant improvements in plant disease detection, particularly in real-time detection. But the above are potential advancements that could be explored to ensure that limitations such as the unavailability of real-time datasets, especially in the African context, biases in existing datasets and real-time models are addressed to improve models for real-time deployment.

### 6.1. Datasets for African Crops Under Natural Conditions

Developing large, diverse, and balanced annotated datasets that capture real-world environmental variability, including different lighting conditions, plant stages, and overlapping disease symptoms, especially in an African context, is vital. Most existing datasets, such as PlantVillage and Kaggle, are collected under controlled conditions with uniform lighting, clean backgrounds, and minimal occlusions. This is only effective for benchmarking models that will be applied in controlled conditions. When it comes to the deployment in real-time environments, they fail to capture the variability of real-world agricultural environments, including occlusions, background artefacts and high-quality cameras.

Future research should focus on developing large-scale, balanced, and diverse real-time datasets that can be integrated with existing datasets such as PlantVillage and Kaggle. Capturing those images at multiple growth stages and co-occurring diseases based on their region in their country, such as how most Indian researchers acquire their own datasets to ensure region-specific solutions. This would also enhance the generalisability and robustness of the model for deployment in agricultural environments. Although image acquisition for such tasks would be time-consuming, it would also ensure that future research does not experience a decrease in robustness and generalizability in real-time environments.

### 6.2. Biases in Datasets

Many datasets exhibit class imbalance, with some diseases being overrepresented while others remain underrepresented. This can lead to biased models that perform well on frequent classes but poorly on rare or visually similar diseases. A proposed solution is to incorporate data augmentation and synthetic image generation, such as Deep Convolutional Generative Adversarial Networks (DCGANs). This technique generates realistic images by training its generator network to create synthetic images and the discriminator network to distinguish synthetic images from original images.

Researchers would apply this technique to the underrepresented image class, which is normally images from a natural environment. This technique will also introduce a wider range of visual representation, thus addressing the class imbalance in the dataset that often leads to model overfitting. This approach will be most effective for real-time datasets, as the networks will learn the underlying distribution of real images and produce synthetic images with similar characteristics, thus reducing the need for extensive manual data collection.

In addition to DCGANs, basic augmentation techniques, such as image rotation, flipping, and colour transformation, can enhance dataset variability and improve the models’ robustness. Another crucial component in improving model generalisation, which allows disease detection systems to recognise trends across species and remain resilient in a variety of environmental circumstances.

The drawback of this technique is that it requires time for training, and if not carefully trained, it might generate repetitive images, thus defeating the purpose of the model design. To mitigate this, careful parameter tuning is required to ensure a balanced and generalizable classification model.

### 6.3. Real-Time Models for Disease Severity Grading, Not Just Detection

Traditional CNN architectures are typically too heavy to be used effectively on low-power edge devices, and their large inference time might make it hard to make quick decisions in the field. Recent studies have investigated the application of real-time object detection algorithms, notably the You Only Look Once (YOLO) series, specifically YOLOv3 and its subsequent versions (YOLOv4, YOLOv5, and YOLOv7), in conjunction with Convolutional Neural Networks (CNNs) for feature extraction and classification. YOLOv3 is an ideal option for agricultural uses that need to be performed rapidly since it strikes an optimal balance between speed and precision. Combining these two architectures not only balances speed and precision but also enables both localization and severity classification, especially for deployment in controlled environments.

The introduction of ViT for models to be deployed in natural environments would also assist in ensuring that even global features and pixel relationships are considered for robust disease and severity classification. But the drawback with traditional ViTs is their computational inefficiency and their requirement for large image datasets for accurate disease detection, which ultimately results in extensive training time, making them impractical for time-sensitive agricultural applications.

To overcome these limitations of traditional ViTs, researchers have explored lightweight and hierarchical ViTs such as the Swin Transformer and GreenViT, which have the ability to reduce computational complexities via parameter reduction whilst maintaining the benefits of global feature modelling. These improvements have been proven to enhance the generalizability of real-time models used for severity classification.

Integrating lightweight ViTs and CNNs with YOLO increases the system’s ability to learn hierarchical characteristics—both local and global features—in real-time environments, allowing for more accurate illness severity classification within detected regions. Furthermore, YOLOv5 and YOLOv7 improve performance by reducing model size and increasing inference speed, which is critical for mobile and drone-based disease monitoring platforms.

By advancing real-time models that are both accurate and efficient, researchers can enable practical, on-field disease detection solutions that support proactive crop management and sustainable farming practices.

### 6.4. Lack of Standardised Benchmarks

A major limitation in evaluating and comparing plant disease detection models is the absence of standardised benchmarks for datasets, metrics, and deployment scenarios. Current studies often use custom datasets with varying image resolutions due to different camera lenses used, image class distributions, and environmental conditions, as the datasets are from a natural environment, which makes it difficult to fairly assess and reproduce results across different models and methods.

Additionally, evaluation metrics vary widely; some researchers report only accuracy, while others sometimes include precision, recall, F1-score and sometimes inference speed. This makes it difficult to benchmark real-time plant disease detection models. There is also limited consistency in measuring real-time performance, such as latency on edge devices or robustness under natural lighting and occlusion in varying regions.

To address this, future work should focus on the following:

- Developing standard benchmarking protocols that define evaluation datasets, metrics, and hardware constraints (e.g., mobile phones or Raspberry Pi technologies).
- Development of an open-source benchmarking tool for fair and reproducible comparison of ML/DL/ViT-based models across different agricultural scenarios. Ensure that these are outlined in the research papers to increase the encouragement of transparency for future real-time models and improvements for existing models.
- Encouraging collaboration across research institutions, particularly in Africa and other underrepresented regions, contributes to a shared benchmarking ecosystem with diverse, annotated, real-world datasets.

Establishing such standards will foster more meaningful comparisons, accelerate innovation, and guide the development of reliable plant disease detection systems that can be deployed in real-world farming environments.

## 7. Conclusions

This paper presents a comprehensive review of popular methods and their pitfalls in the domains of plant disease detection, focusing on the spectrum of ML and DL. Most researchers have developed models that have improved precision agricultural practices and reduced crop losses.

SVM, kNN, decision trees, Random Forest, and other classical ML models have shown success in early implementations, especially when combined with selective datasets and handmade features. Their sensitivity to environmental variability and reliance on feature engineering, however, continue to hinder their effectiveness in real-world scenarios such as agricultural settings. DL models have become extremely effective tools that can directly learn complicated patterns from raw picture data, especially CNN-based architectures like AlexNet, ResNet, and YOLO. In terms of accuracy and generalisation, these models perform better than conventional ML algorithms, particularly when combined with properly pre-processed data and suitable augmentation methods. YOLO variations are notable for their speed and versatility when used with edge devices in real-time applications.

Significant limitations still exist despite these developments; this review also discusses those limitations and offers possible solutions. These include the computational difficulties of deploying high-performing models in resource-constrained situations, the class imbalance in available datasets, and the lack of diverse, real-world, properly annotated datasets. The development of reliable real-time lightweight models such as DCGANs, YOLOv3, and GreenViT can be used to resolve dataset constraints by the integration of real-time images to the datasets and enhance model interpretability and generalisation across plant species and settings must be the main goals of future research.

Researchers can help create effective, versatile, and field-ready plant disease detection systems by tackling these issues, which will ultimately support sustainable agricultural innovation, proactive crop management, and food security.

## Figures and Tables

**Table 1:** Pathogens and their symptoms in infected plants [6].

Type of Pathogens	Main Symptom
Virus	Reduced growth in certain parts of the plant or the entire plant, discolouration of the leaves and deformities of plant stems or other organs.
Fungi	Overgrowth, rot, mould, deformation and wilting in the plant organs.
Bacteria	Wilting, chlorosis, rot, overgrowth (galls), and scab display a plant infected by bacteria.

**Table 2:** Comparison of various filters used in the image preprocessing [21].

Filter	Advantages	Disadvantages
Median	Robust, better edge preservation.	Prone to being corrupted by Gaussian noise.
Adaptive median	Reduces impulse response and distortion and smoothens noise in the image.	Reduced data loss compared to the median.
Gaussian	Effective for removing Gaussian noise.	Time-consuming and defective image details.
Weiner	Reduces image noise better than a median filter.	Results are mostly distorted, resulting in blurry images.
Adaptive histogram equalisation.	Overamplifies the noise.	Time-consuming.

**Table 3:** Comparison of various image segmentation and classification techniques [26].

Segmentation Techniques	Contribution	Limitations
Region-based segmentation	More noise immune, works well in homogeneous regions.	Computationally complex and requires more processing time.
Watershed segmentation	Computationally efficient.	Over segmentation,
Edge-based approaches	Works well for images having good contrast.	Less immune to noise, Inaccurate sometimes, complex computation.
K-means segmentation	Tighter clusters than hierarchical methods, particularly if the clusters are globular, are faster.	Prediction of K-value is difficult.
Histogram thresholding	Less computationally complex, does not need prior knowledge.	Does not consider the spatial details.
Neural network approaches	Less complex, high processing speed.	Training time is long, and overtraining is avoided.

**Table 4:** Comparison of various feature extraction techniques [29].

Feature Extraction	Contribution	Limitation
Local Binary Pattern (LBP)	Efficient and simple; good for texture classification.	Sensitive to noise and lighting variations.
PCA	Reduces dimensionality; highlights variance in features.	May lose spatial features; assumes linearity.
Colour Co-occurrence Matrix (CCM)	Captures the spatial distribution of colours in the HSI space	Computationally expensive; less robust to colour variation.
Linear Discriminant Analysis (LDA)	Maximises class separability; good for supervised classification.	Assumes Gaussian distribution; equal covariance.
Partial Least Squares Discriminant (PLS-R)	Handles multicollinearity; suitable for hyperspectral data.	Complex interpretation; sensitive to model configuration.

**Table 5:** Summary of classical ML models used in plant disease detection.

Refs.	Algorithm	Model Description	Plant Under Test	Accuracy (%)	Limitation
[36]	DT	The image dataset undergoes preprocessing and segmentation to extract the relevant plant features. Then the morphological features are fed to the decision tree classifier to identify diseases of the plant from healthy plants.	Tomato, lemon, rose, papaya and banana.	95.26	Potential overfitting, lower interpretability, and delays its rapidness in disease identification.
[37]	NB	Used fuzzy logic to convert expert linguistic knowledge about papaya diseases into numerical values using a Triangular Fuzzy Number (TFN) membership function. The data was classified using NB, with forward chaining applied to enhance inference and improve disease detection accuracy for farmers without expert intervention.	Papaya	88	Assumes feature independence; may not capture complex patterns.
[38]	kNN	The process included resizing images to 256 × 256 pixels, applying histogram equalisation for contrast enhancement, converting RGB images, extracting features using the GLCM, and segmenting leaf regions. The extracted features were classified using the KNN algorithm to identify diseases and evaluate.	Various images from the PlantVillage dataset.	99.96	Computationally intensive; may require large datasets.
[39]	SVM	The technique with non-linear Gaussian kernels was utilised to classify diseased cotton leaves by finding a hyperplane that maximises the distance between each class in an N-dimensional space.	Bacterial Blight, Alternaria, Gray Mildew, Cereospra, and Fusarium wilt.	83.26	Performance varies with kernel choice; it may not scale well with large datasets.
[40]	RF	The model detected diseases in papaya leaves, trained on 160 images taken against plain backgrounds to reduce occlusion. The images were converted to RGB, followed by the HSV technique for histogram equalisation. HOG technique was utilised for feature extraction, and RF for classification on whether the plant was infected or not.	Papaya leaves.	70.14	May be less interpretable; performance depends on the number of trees and depth.

**Table 6:** Performance results of various CNN model architectures.

Refs.	CNN Architecture	Dataset	Number of Images	Plants Under Test	Types of Diseases	Accuracy (%)
[59]	VGG-16	PlantVillage	15,915	19 different classes of tomato, grape, apple and corn.	Late blight, scab, early blight, black spot and rust	95.20
[60]	ResNet50	Kaggle and Mendelay.	Not specified	Paddy leaf	bacterial leaf blight, brown spot, leaf smut and tungro.	97.30
[61]	AlexNet	PlantVillage and GBPUAT	4004	Mango and Potato	Anthracnos, early blight	98.33
[62]	GoogleNet	PlantVillage	3171	Apple	Cedar Apple Rust, Apple Scab, and Black Rot	99.79
[63]	MobileNet and LeNet	Not specified	4508	Rice	Hispa, leaf blight, brown spot and tungro	MobileNet: 96.10 LeNet: 90.20
[64]	MobileNetV2	Own dataset	7800	Cucumber leaf	Not specified	90.38

**Table 7:** Performance of different CNN model architectures for the identification of diseases on the testing dataset [3].

Model	Accuracy Rate (%)	Average Error	Time(s/Epoch)
AlexNet	98.64	0.0658	1022
AlexNetOWTBn	99.07	0.0332	1125
GoogleNet	97.06	0.0984	2670
Overfeat	98.26	0.0848	1570
VGG	98.87	0.0542	4208

**Table 8:** Comparison and relevance of each single-stage algorithm in plant disease detection.

Algorithm	Speed	Accuracy	Strengths	Weaknesses
YOLOv3/v4 [66]	Very fast	High	Real-time inference, general-purpose	May miss small lesions
SSD [71]	Fast	Moderate–High	Multi-scale detection, lightweight	Less accurate than RetinaNet
RetinaNet [70]	Moderate	Very High	Handles class imbalance, detects small objects	Slower, higher computational cost

**Table 9:** A comparative evaluation of the proposed GreenViT model’s frames per second (FPS) performance against various DL models is presented [80]. This analysis illustrates the relative inference speed of each model. Downward arrows (↓) indicate that lower values are preferable, whereas upward arrows (↑) signify that higher values are desirable, depending on the metric being assessed.

Model	Parameters (M) ↓	Size (MB) ↓	FPS ↑	
			RPi 4B+	CPU
VGG19	200.25	229.0	0.47	9.49
VGG16	147.15	168.0	0.62	11.09
EfficientNetB0	4.05	46.9	2.69	19.74
MobileNetV1	3.23	37.1	8.23	22.96
MobileNetV3Small	1.53	18.0	7.43	27.94
Vit Base	86.00	345.0	0.21	19.83
GreenViT	21.65	247.0	0.34	22.19

**Table 10:** Model performance of ViT based architectures in diverse agricultural settings.

Refs.	Dataset	Model	Deployment Technique	Deployment Environment	Accuracy	Model Suitability
[81]	Own images	Transformer-based multimodal fusion framework	Smartphone mobile application	Real-time natural environment	94%	High robustness and generalisation.
[82]	Multispectral plant dataset	Hybrid ViT–CNN (ViT_r50_132 and ViT_r26_s32 with ResNet50)	Local computing device	Field conditions (with NIR)	88.86%	Improved accuracy; challenges with complexity and overfitting
[85]	Custom tomato leaf dataset	EfficientNetV2 and Swin Transformer networks	On-device (optimised for speed)	Mixed natural and low-light environment	99.70%	High accuracy and generalizability; well-suited for real-time deployment
[86]	Rose leaf images from (Kaggle dataset)	ViT-B/16 (Baseline Vision Transformer)	Local computing device	Controlled lab environment	93%	Excellent accuracy in ideal conditions; limited real-world generalisation.

## Data Availability

No new data were created in this study.
